# Dimensional crossover and cold-atom realization of topological Mott insulators

**DOI:** 10.1038/srep08386

**Published:** 2015-02-11

**Authors:** Mathias S. Scheurer, Stephan Rachel, Peter P. Orth

**Affiliations:** 1 Institute for Theory of Condensed Matter, Karlsruhe Institute of Technology (KIT), 76131 Karlsruhe, Germany; 2 Institute for Theoretical Physics, TU Dresden, 01062 Dresden, Germany

## Abstract

Interacting cold-atomic gases in optical lattices offer an experimental approach to outstanding problems of many body physics. One important example is the interplay of interaction and topology which promises to generate a variety of exotic phases such as the fractionalized Chern insulator or the topological Mott insulator. Both theoretically understanding these states of matter and finding suitable systems that host them have proven to be challenging problems. Here we propose a cold-atom setup where Hubbard on-site interactions give rise to spin liquid-like phases: weak and strong topological Mott insulators. They represent the celebrated paradigm of an interacting and topological quantum state with fractionalized spinon excitations that inherit the topology of the non-interacting system. Our proposal shall help to pave the way for a controlled experimental investigation of this exotic state of matter in optical lattices. Furthermore, it allows for the investigation of a dimensional crossover from a two-dimensional quantum spin Hall insulating phase to a three-dimensional strong topological insulator by tuning the hopping between the layers.

Experiments of cold atoms in optical lattices allow addressing fundamental questions of condensed matter physics^1^,^2^. From a condensed matter perspective, those experiments accomplish two major achievements. First, they can serve as quantum simulators of systems that have a solid-state analogue with the crucial difference that one has a high degree of control and excellent tunability of most of the system parameters^3^. One may even go beyond traditional models and study parameter regimes that are not accessible in the solid state. On the other hand, cold-atom setups provide platforms for the realization of novel phases of matter and new phenomena that have never been observed in the solid state so far. Examples of the latter is the interaction driven Mott-superfluid quantum phase transition in the Bose-Hubbard model^4^ or SU(N) magnetism using alkaline-earth atoms^5^. Examples of the first case are investigations of Fermi-Hubbard models^6^,^7^,^8^,^9^,^10^ in search for *d*-wave superfluidity or the study of three-dimensional Rashba spin-orbit coupling^11^.

One of the most active areas of research in condensed matter physics at present are topological phases and, in particular, topological insulators^12^,^13^,^14^. These phases represent a new paradigm of quantum matter with a bulk gap but gapless surface states that are protected by symmetries such as time-reversal invariance. In real materials, the non-trivial band topology is usually caused by virtue of spin-orbit coupling. The realization of synthetic spin-orbit couplings for cold atoms^15^,^16^,^17^,^18^,^19^,^20^,^21^,^22^,^23^,^24^,^25^ thus marks the advent of topology to the field of ultra-cold gases.

The dimensionality of space is an important parameter of topological phases in their classification according to anti-unitary symmetries^26^. The crossover between different dimensions is in general non-trivial: simply stacking the two-dimensional (2D) 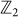 topological insulator^27^,^28^,^29^,^30^ does not lead to its 3D counterpart, the strong topological insulator (STI)^31^,^32^,^33^. Instead, one obtains a 3D weak topological insulator (WTI)^34^.

The situation becomes even more interesting in the presence of sufficiently strong interactions, where the topological classification^26^ breaks down in some cases (see, *e.g.*, Ref. 35) and new phases can appear. As the interaction strength in cold-atom systems can be tuned over a wide range, they provide an ideal testbed to study the interplay of non-trivial topology and strong interactions. One of the most exciting theoretical proposals for such exotic states of matter is the *topological Mott insulator* of Pesin and Balents^36^. It represents a spin liquid-like phase where the charge is stripped from the original electrons and frozen in a Mott insulating phase; the spinons (*i.e.*, the electrons' spin degree of freedom), however, inherit the non-trivial band topology of the topological insulator and provide spin-only gapless surface states. The experimental observation of this state has so far remained elusive and even the theoretical understanding is rather limited^36^,^37^,^38^,^39^,^40^. Note that this notion of a topological Mott insulator, which will be used in this paper, crucially differs from that of the proposal in Ref. 41 (see Supplementary Discussion S1).

Here, we propose an experimental setup which represents an example of both achievements of cold atoms in optical lattices: first, a quantum simulator for known phases which can be tuned into each other in a unique way. Second, a realization of exotic weak and strong topological Mott insulating phases which have not yet been found in solids.

The model we propose is experimentally feasible and can be seen as a 3D generalization of an experimental setup for 2D topological insulators in optical lattices^42^,^43^,^44^,^45^,^46^. We show that the 2D system, which effectively consists of two time-reversed copies of massive Dirac Hamiltonians, can be tuned to a 3D weak or strong topological insulator just by varying a single hopping parameter. Since on-site Hubbard interactions are experimentally available, we analyze the interacting phase diagram within slave-rotor theory^47^,^48^,^49^. Interactions drive a transition into a Mott insulating state, where the topological nature of the system survives and emerges as fractionalized gapless spin-only surface modes. Our proposal for a cold atom experiment and its possible subsequent realization will help to establish a deeper understanding of this exotic phase.

## Results

### Dimensional crossover from 2D to 3D topological insulator

The starting point of our analysis is the time-reversal invariant Hofstadter model on the square lattice^42^,^43^,^44^

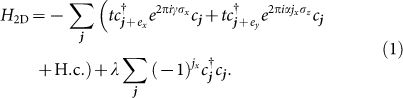
It describes fermions with spin-1/2 in the presence of synthetic gauge fields; the parameter *α* describes the flux per plaquette of an artificial magnetic field perpendicular to the 2D plane, which due to the Pauli matrix *σ_z_* points in opposite directions for opposite spins. The spin mixing term *γ* induces spin flips if the particle moves along the *x*-axis, and the *λ*-term describes a staggering of the optical lattice potential along the *x*-direction. All three terms can be experimentally realized^42^,^43^,^44^.

**Figure 1:**
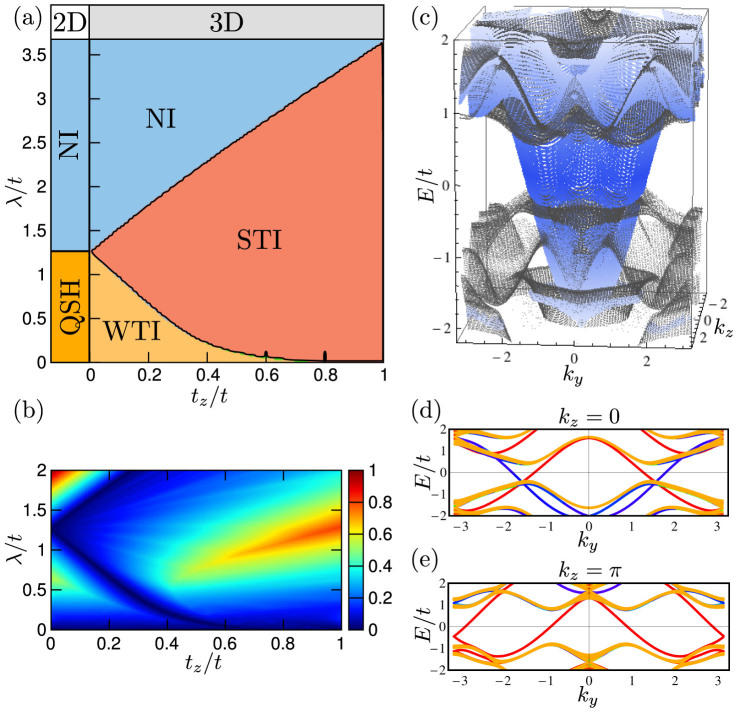
Phase diagram and spectra of the lattice model. (a) 2D–3D crossover phase diagram as a function of layer coupling *t_z_* and staggered lattice potential *λ*. (b) Bulk gap as a function of *t_z_* and *λ*. (c) Surface state spectrum (blue) of the isotropic 3D system *t_z_* = *t*, *λ* = *λ_c_* at the *x* = 0 surface as a function of momenta *k_y_* and *k_z_*. Bulk states are shown as gray dots. We use open (periodic) boundary conditions along *x* (*y*, *z*). (d–e) One-dimensional cuts of the spectrum for fixed values of *k_z_* = 0 and *k_z_* = *π*. Gapless edge states at *x* = 0 (*x* = *L*) edge are shown in blue (red) yielding *z*_0_ = 1 and *z_π_* = 0 and thus *ν*_0_ = *z*_0_ + *z_π_* = 1.

The model belongs to the symmetry class AII^26^ and its phase diagram in 2D hosts quantum spin Hall (QSH) and normal insulating (NI) as well as (semi-)metallic phases^42^,^45^,^46^. The QSH phase is characterized by an odd number of helical edge states per edge, while the NI features an even number (including zero). The topological 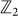 invariant *ν* distinguishes between QSH (*ν* = 1) and NI (*ν* = 0) phase^28^. It is sufficient to consider half-filling and fixed values of *α* = 1/6 and *γ* = 1/4, as the phase diagram as a function of the staggered potential *λ* already contains both gapped phases as well as semi-metallic points. As shown in Fig. 1(a) the 2D system is a QSH insulator for |*λ*| < *λ_c_* = 2^1/3^*t* and becomes normal insulating for |*λ*| > *λ_c_*^42^,^45^,^46^. The system is semi-metallic at *λ* = 0 hosting two doubly degenerate Dirac cones and at *λ* = *λ_c_* with one doubly degenerate Dirac cone.

We are interested in studying the dimensional crossover from two to three dimensions by continuously turning on a hopping parameter *t_z_* in the third direction that couples the different 2D layers. The easiest interlayer coupling term that makes STI phases possible is of the form 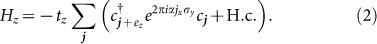
It contains a synthetic gauge field that represents an artificial magnetic field along the *y*-direction, which points in opposite directions for spins aligned parallel and anti-parallel to the *y*-axis. Such a term can be most easily implemented in an all optical realization of the lattice potential^43^,^44^. Note that this term respects time-reversal symmetry and, hence, the resulting 3D Hamiltonian *H*_2D_ + *H_z_* still belongs to class AII.

As we have to deal with a 12-band model in 3D, some intuitive understanding is desirable. For this reason, we derive an effective theory valid in the vicinity of the point (*t_z_*, *λ*) = (0, *λ_c_*) in the phase diagram of Fig. 1(a), where all the distinct phases meet: QSH and NI (when *t_z_* = 0) as well as STI, WTI, and NI (when *t_z_* > 0). The doubly degenerate Dirac cone at this multi-critical point is formed out of four bands, the other eight bands are well-separated from the Fermi level.

Neglecting corrections quadratic in *t_z_*/*t*, (*λ* − *λ_c_*)/*t*, and *k_x_*, *k_y_*, quasi-degenerate perturbation theory^50^ yields the effective four-band Hamiltonian 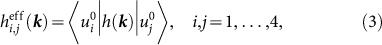
where *h*(***k***) denotes the full 12-component Bloch-Hamiltonian associated with the terms in Eqs. (1) and (2), 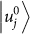 are the four zero-energy eigenfunctions of *h*(**0**) at the multi-critical point. Retaining only the relevant terms linear in *k_x_* and *k_y_*, Eq. (3) assumes the form 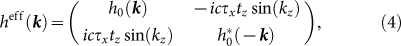
where the upper left 2 × 2-block is given by
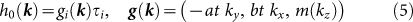
with mass 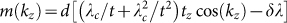 and *δλ* = *λ* − *λ_c_*. The Pauli matrices *τ_i_* act within the 2 × 2-blocks in Eq. (4) and *a*, *b*, *c*, *d* are positive constants. For convenience, we have chosen the basis functions such that time-reversal is given by 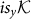, where the Pauli matrices *s_i_* act between the different 2 × 2-blocks and 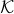 denotes complex conjugation. The relation between the lower and the upper diagonal blocks in Eq. (4) is thus a consequence of time-reversal symmetry. In addition, this convention directly reveals the connection of the 2D system (*t_z_* = 0) to the Bernevig-Hughes-Zhang-model^29^, the paradigmatic model of a QSH state. Consequently, we immediately know that *δλ* = 0 marks the boundary between QSH and NI phases. As the regime *δλ* > 0 is adiabatically connected to the topologically trivial limit *λ* → ∞, the effective model reproduces the 2D phase diagram.

To continue with the 3D system, let us first emphasize that the effective Hamiltonian (4) is valid for the entire range −*π* < *k_z_* ≤ *π*, since *t_z_* (and not *k_z_*) has been taken as expansion parameter. In 3D, insulating phases are characterized by four 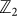 invariants *ν_i_*, with *i* = 0, 1, 2, 3, defined by invariants of 2D cuts of the 3D Brillouin zone^31^,^32^,^33^. To determine the strong invariant *ν*_0_ ≡ (*z*_0_ + *z_π_*) mod 2, we need to calculate the 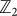 invariants *z*_0_ and *z_π_* associated with the time-reversal invariant planes *k_z_* = 0 and *k_z_* = *π*. As Eq. (4) again assumes the form of the Bernevig-Hughes-Zhang-model for fixed *k_z_* = 0 and *k_z_* = *π*, we can directly conclude that the two QSH invariants *z*_0_ and *z_π_* differ when *m*(0)*m*(*π*) < 0. Therefore, the system is in the STI phase if and only if 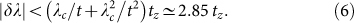
Note that, despite the fact that the mass *m*(*k_z_*) has to vanish somewhere between *k_z_* = 0 and *k_z_* = *π*, the Hamiltonian is still fully gapped. This is a consequence of the block off-diagonal terms in Eq. (4) that are finite for *k_z_* ≠ 0, *π*. Interestingly, for *λ* = *λ_c_*, this model allows for a transition into an STI, a phase that has no 2D analog, already for infinitesimal coupling *t_z_*.

Being adiabatically connected to *t_z_* = 0, the other two phases (|*δλ*| > 2.85 *t_z_*) can easily be identified from the knowledge about the two-dimensional system. For *δλ* > 2.85 *t_z_*, we find a NI, whereas, in case of *δλ* < −2.85 *t_z_*, the system resides in a WTI phase characterized by (*ν*_0_; *ν*_1_, *ν*_2_, *ν*_3_) = (0; 0, 0, 1)^31^,^32^,^33^.

Finally, the effective Hamiltonian also allows understanding why a spin- and position-independent hopping term along the *z*-direction cannot result in an STI phase. Such a term would simply lead to a contribution proportional to the identity matrix in the 12-band Bloch-Hamiltonian *h*(***k***) and thus to a term 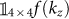 in the effective low-energy theory (3). Consequently, ***g*** in Eq. (5) would be independent of *k_z_* and hence *z*_0_ = *z_π_*, excluding the appearance of an STI.

We verified our analysis of the effective model by numerically computing the 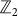 invariant in all insulating phases of the full 12-band model using the approach of Ref. 51. The corresponding phase diagram is illustrated in Fig. 1(a) and is in perfect agreement with our previous discussion. The 2D gapped QSH (NI) phase turns into a WTI (NI), but most importantly the STI phase emerges from the 2D quantum critical point. Note that this is the only possibility for an STI to appear at infinitesimal coupling *t_z_* as this phase is not adiabatically connected to the NI and QSH.

An alternative way of realizing a dimensional crossover in topological systems consists of varying the thickness in the *z*-direction. As discussed in Refs. 52 and 53 in the solid state context, this leads to oscillatory properties of the effective 2D system. In contrast, in our crossover scheme, the system is fully 3D at every finite *t_z_* which is more feasible in a cold atom experiment. For that reason we calculate 3D topological invariants for any *t_z_* ≠ 0, which do not show oscillatory behavior.

The bulk gap, which closes at the phase transitions, is shown in Fig. 1(b). It reaches its maximal value of the order of *t* for isotropic hopping. Computing the spectrum with open (periodic) boundary conditions along the *x*- (*y*, *z*)-direction, we clearly observe in Fig. 1(c) (shown for *t_z_* = *t*) a single gapless Dirac-like surface state (in blue) localized at the *x* = 0 surface, which crosses the bulk gap (bulk bands in gray). The Dirac point is located inside the bulk bands (which is not unusual). The surface Fermi circle encloses an odd number of time-reversal invariant points reflecting the non-trivial strong invariant *ν*_0_ = 1. One-dimensional cuts of the spectrum for *k_z_* = 0 and *k_z_* = *π* are shown in Fig. 1(d) and (e), respectively. We see explicitly that *m*(0) and *m*(*π*) have opposite signs as the parity of the number of Kramers partners of surface states on a given boundary changes from odd (QSH) to even (NI) when *k_z_* is tuned from 0 to *π*.

### Interaction effects and topological Mott phases

**Figure 2:**
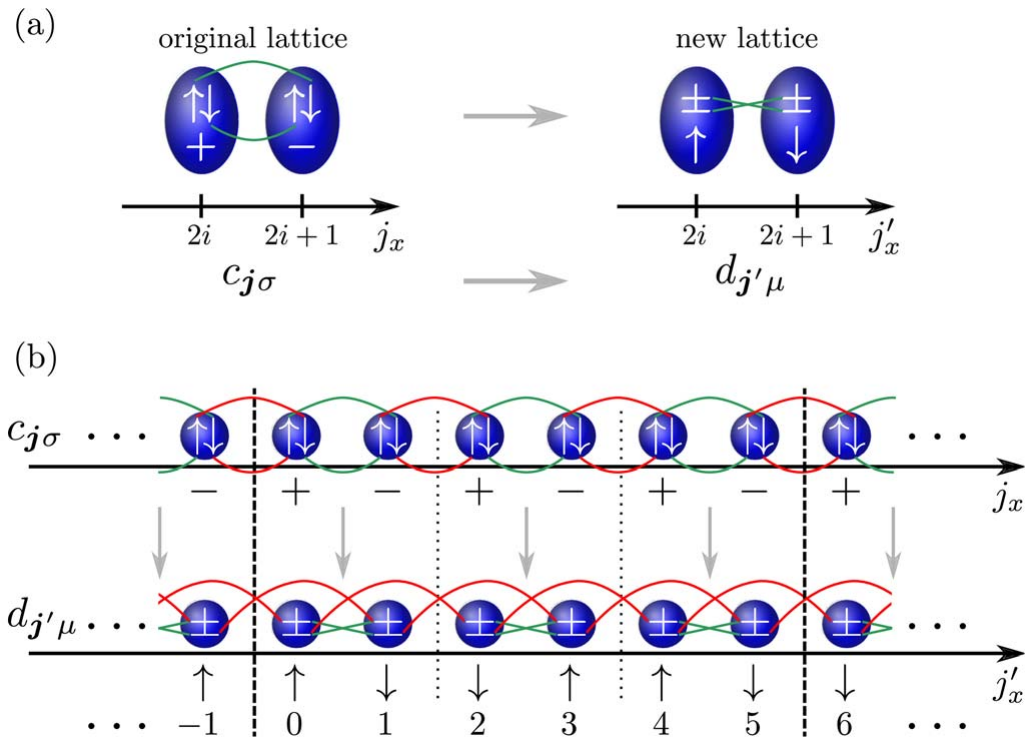
Twisting the Hubbard interaction. Part (a) shows the relabeling of the spin *σ* and spatial even/odd site *μ* degrees of freedom. To obtain an identical Hamiltonian *H*_2D_ + *H_z_* one must realize different hopping elements between nearest and next-nearest neighbor sites as illustrated in (b) for the *x*-direction. The required hoppings along the *y*- and *z*-direction are described in detail in Supplementary Discussion S2.

While non-interacting topological phases are characterized by the properties of their Bloch functions and can be classified according to symmetries, the question what occurs in the presence of interactions is of great current interest^54^,^55^,^56^,^57^. However, there are still many open questions concerning the fundamental classification of interacting states of matter and the explicit investigation of concrete interacting topological systems. So far, in explicit calculations, mainly the effect of Hubbard on-site interactions 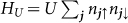, where 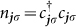, has been considered in order to address interaction effects in topological band structures^36^,^37^,^58^,^59^. While this is very natural for solid state systems, the achievement of ultracold quantum gases and optical lattices allows considering interactions which cannot be realized in real materials, opening a path towards exotic physics and giving rise to an even richer phenomenology. As illustrated in Fig. 2(a), the key idea for effectively realizing an exotic interaction term is to encode the spin degree of freedom *σ* = ↑, ↓ spatially and use the internal atomic hyperfine states to represent the even/odd site information along *x*: *μ* = + for *j_x_* = 2*n* and *μ* = − for *j_x_* = 2*n* + 1 with integer *n*. We denote the fermionic operators of the new lattice by 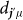.

As one can readily see in Fig. 2(a), a local Hubbard interaction realized on the new lattice, 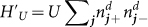, where 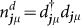, reads, at half-filling, in terms of the occupation numbers of the original *c****_j_***_*σ*_ fermions as
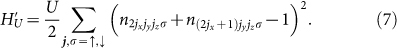
Consequently, 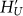 does not couple ↑-spin and ↓-spin on a given site but instead pairs of neighboring sites having the same spin orientation; the Hubbard term is twisted. As shown below, it will generate topological Mott insulating (TMI) phases^36^ if we keep the same non-interacting Hamiltonian *H*_2D_ + *H_z_* as before. To achieve this, one needs to experimentally implement different laser induced hopping elements for the 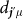 fermions as illustrated in Fig. 2(b) for the *x*-direction. Only nearest neighbor and next-nearest neighbor terms along the three spatial directions are required.

The TMI is characterized by a fractionalization of the original atoms into an internal and a number degree of freedom. In the TMI phase the atoms are localized, but their internal degree of freedom remains deconfined and inherits the non-trivial band topology of the original fermions. This state has been proposed to occur in Ir-based pyrochlore materials^36^, but has so far never been experimentally observed.

We approach the TMI using slave-rotor theory^47^,^48^,^49^ which starts by writing the fermion operator as a product of number and internal degree of freedom 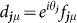. Here, *θ****_j_*** denote phases conjugate to the total particle number (of *d****_j_***_*μ*_ fermions) on site ***j*** and *f****_j_***_*μ*_ is a spinon fermion operator that carries the internal index. The system is then described by two coupled mean-field Hamiltonians: a 3D quantum XY rotor model, which captures the number degrees of freedom, and a renormalized non-interacting spinon Hamiltonian (see Methods). As the strength of the quantum fluctuations in the rotor model is determined by the interaction *U*, the rotor undergoes a transition from a ferro- to a paramagnetic state as *U* is increased beyond a critical value *U_c_*. This transition corresponds to the Mott transition. The spinons, on the other hand, are characterized by a bandstructure with renormalized parameters (set by the correlations between rotors), that can be topologically non-trivial and carry gapless surface excitations.

**Figure 3:**
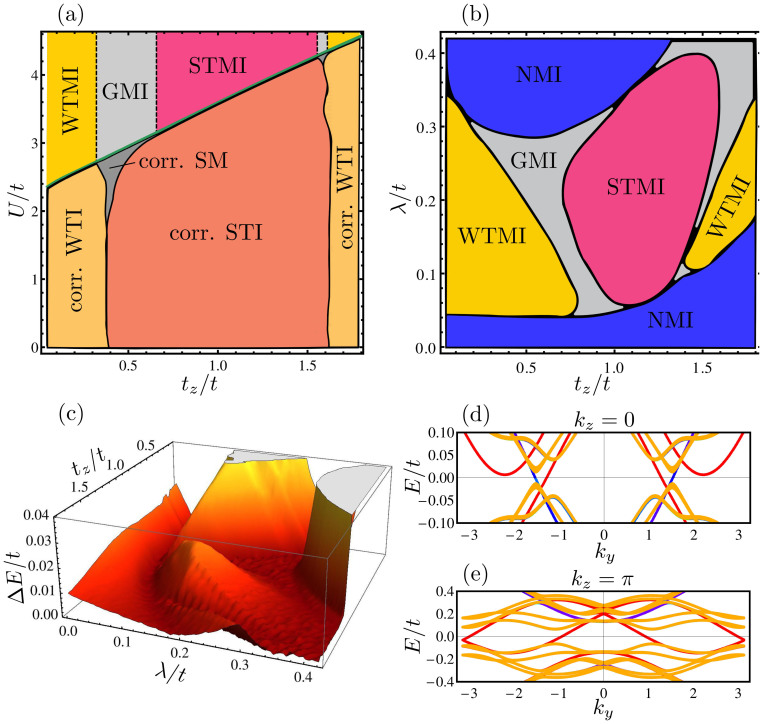
Properties of the interacting system according to slave-rotor theory. (a) Interacting phase diagram as a function of interaction *U* and hopping *t_z_* for fixed *λ*/*t* = 0.25. Upon increasing *U* the phases found at *U* = 0 remain mostly intact with renormalized parameters. For 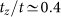 an extended correlated semi-metallic (SM) phase appears at 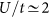. At a critical value *U_c_*/*t* (green line) the system enters a Mott insulating (MI) state with weak and strong topological MI (WTMI and STMI) as well as gapless MI (GMI) phases. The GMI phase exhibits a semimetallic spinon spectrum. Dashed lines are a guide to the eye showing that Mott phases persist for *U* > *U_c_*. (b) Interacting phase diagram and (c) bulk gap at *U_c_* as a function of staggering *λ* and hopping *t_z_*. The STMI phase occupies a large part of the phase diagram and features a significant bulk gap. (d–e) One-dimensional cut through the spinon bandstructure in the STMI phase for the isotropic system *t_z_* = *t* and *λ*/*t* = 0.15. We use open (periodic) boundary conditions along *x* (*y*, *z*). Bulk states are shown in yellow, gapless spinon edge states at the *x* = 0 (*x* = *L*) edge are shown in blue (red).

Analyzing the interaction term (7) within slave-rotor mean-field theory, we find that the interaction only renormalizes the hopping elements *t*, *t_z_* but leaves *λ* unchanged. Physically, this is due to the fact that 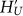 is not sensitive to an imbalance between *μ* = + and *μ* = −. Furthermore, the interaction induces slight spatial anisotropies between the hopping elements as well as inhomogeneities in the six atomic unit cell. In Fig. 3(a) we show the interacting phase diagram as a function of *U*/*t* and *t_z_*/*t* in the region of the dimensional crossover for *λ*/*t* = 0.25. The hoppings of the *d****_j_***_*μ*_ fermions on the new lattice have been chosen such that their Bloch Hamiltonian is unitarily equivalent to the Bloch Hamiltonian of the *c****_j_***_*σ*_ fermions and, consequently, the *U* = 0 line reproduces a cut of the phase diagram in Fig. 1(a). The non-interacting phases remain stable for small interaction 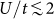, but the anisotropies lead to the emergence of a correlated semi-metallic (SM) phase at intermediate 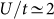, where interactions induce a formation of Dirac cones.

Stronger interactions drive the system across a quantum phase transition to various Mott phases at a critical interaction strength *U_c_*. In the Mott state, the fermionic degrees of freedom are fractionalized with charge (or number) and internal hyperfine degrees of freedom being split. While the number degrees of freedom are localized, the internal hyperfine degrees of freedom are deconfined. The resulting spinon bandstructure can remain topological across the Mott transition, which defines the sought after strong and weak topological Mott insulator (STMI and WTMI) phases. The dashed lines in Fig. 3(a) are only a guide for the eye indicating that the different Mott phases persist for 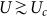. At even larger interaction strength magnetically ordered phases are likely to emerge (not shown). Note that the various complex hopping terms lead to a rather frustrated spin exchange and we expect the onset of magnetism for 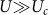.

In Fig. 3(b) we present the interacting phase diagram at the critical interaction strength *U_c_* as a function of *λ*/*t* and *t_z_*/*t* to show that WTMI and STMI occupy a large part of it. These phases exhibit a bulk gap of the order of 10% of the renormalized bandwidth (see Fig. 3(c)). Due to the topological nature of the spinon bandstructure, they feature gapless spinon surface states shown in Fig. 3(d–e) which is the defining property of the TMI phases.

## Discussion

In our proposal, time-reversal is a non-local operation interchanging sites with even and odd *j_x_* in the new basis of *d****_j_***_*μ*_ fermions defining the experimental lattice. Clearly, this is a consequence of the fact that the transformation 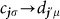 depicted in Fig. 2 is non-local. However, on the scale of the system size, time-reversal and the mapping to the new lattice can be regarded as quasi-local operations and, in particular, do not interchange surface states at distinct surfaces. Thus any quasi-local perturbation of the 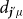 fermions is also quasi-local in the basis of *c****_j_***_*σ*_ and the surface states in the new lattice localized on a given boundary form a time-reversal symmetric electronic system. This proves the protection^12^ of the surface states against localization by time-reversal symmetric disorder.

Let us now discuss how the TMI phases are reflected in observable quantities. The atoms are frozen in a Mott insulating state which can be detected via standard time-of-flight measurements. The most straightforward way to detect the spinon surface states is to measure the spin-dependent spectral function. Alternatively, one might consider transport or thermodynamic quantities^60^,^61^. In contrast to the non-interacting topological insulators, the TMIs do not exhibit a finite “charge” (or number) response at low frequencies due to the Mott gap. The gapless spinon surface states, however, carry entropy resulting in a thermal conductivity that is linear in temperature^36^ and a heat capacity that scales as *T* ln(1/*T*)^38^. Distinguishing between WTMI and STMI is possible as they feature a different number of surface states. The specific heat and thermal conductivity will thus be quantitatively different in the two phases.

To conclude, we have presented an experimentally feasible tight-binding Hamiltonian which allows performing a quantum simulation of a dimensional crossover from a 2D quantum spin Hall to 3D strong and weak topological insulator phases. Considering the effect of on-site interactions, we have shown that weak and strong topological Mott insulator phases can be realized. We are confident that our results will lead to a subsequent experimental realization of this exotic state of matter.

## Methods

To derive the interacting phase diagrams reported in the main part of the paper, we apply slave-rotor theory^47^,^48^,^49^ to the Hamiltonian 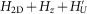 restated in the new basis (*d****_j_***_*μ*_ fermions) where the interaction is an on-site Hubbard term. Within this approach, one introduces phases *θ****_j_*** conjugate to the total number of fermions on one site of the lattice and auxiliary fermionic operators *f****_j_***_*μ*_ via
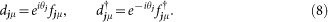
To restrict the theory to the physical part of the Hilbert space, we have to impose the constraint
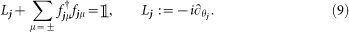
This condition will be treated on average and accounted for by introducing Lagrange multipliers *h****_j_***. Passing to a path-integral description, the associated action is given by
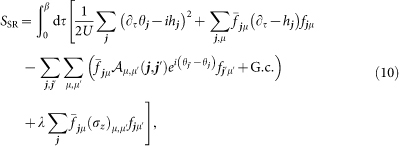
where *f****_j_***_*μ*_, 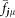 denote Grassmann variables and 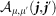 represent the hopping matrix elements in the new lattice.

In this work, we use the sigma-model description of Ref. 47, where the phase degrees of freedom are represented by complex bosonic fields
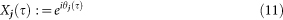
with the nonlinear constraint
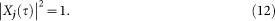
The latter will be ensured on average via additonal Lagrange multipliers *ρ****_j_***. Applying a mean-field approximation to the hopping terms in Eq. (10), we arrive at the bosonic and fermionic actions
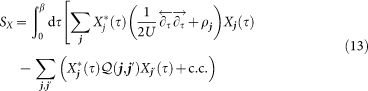
and
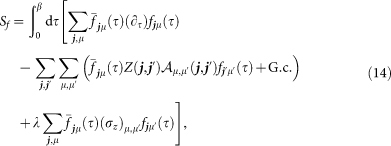
that are coupled via the self-consistency equations
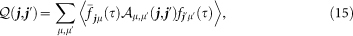

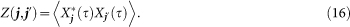


Here we have already taken into account that, at half-filling, the constraint (9) is satisfied on average by choosing *h****_j_*** = 0. Note that only three of the Lagrange multipliers *ρ****_j_***, *e.g.* *ρ*_0,0,0_, *ρ*_2,0,0_ and *ρ*_4,0,0_, are independent which is due to the combination of translation symmetry and time-reversal invariance.

For any given *U* one has to solve the set of equations (15, 16) and find the correct Lagrange multipliers *ρ*_0,0,0_, *ρ*_2,0,0_, *ρ*_4,0,0_ yielding the renormalization factors *Z*(***j***, ***j***′) of the auxiliary fermions *f****_j_***_*μ*_. The band structure resulting from Eq. (14) can again be classified exactly as for non-interacting fermions leading to the variety of different correlated topologically trivial and non-trivial phases discussed in the main part of the paper. The transition into the Mott phase, where the local number degree of freedom is frozen out, occurs when the gap of the bosons in Eq. (13) closes. This point marks the transition from a ferromagnetic to a paramagnetic state of the rotors *X****_j_***. Even in the paramagnetic state there exist short range correlations between the rotors which implies that the renormalization factors *Z*(***j***, ***j***′) are non-zero. A topologically non-trivial spinon bandstructure while having paramagnetic rotors defines the weak and strong topological Mott insulator phases.

To obtain the phase diagram in Fig. 3(b) in the main text, we have calculated the renormalization factors (16) right at the Mott transition (*U* = *U_c_*) and determined the topological invariant of the auxiliary fermions *f****_j_***_*μ*_ using the numerical approach of Ref. 51. The phase boundaries for 0 < *U* < *U_c_*, shown in Fig. 3(a) in the main text, have been obtained via linear interpolation of the renormalization factors, 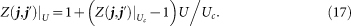


## Author Contributions

M.S.S., S.R. and P.P.O. contributed extensively to the calculations, prepared the figures and wrote the paper.

## Supplementary Material

Supplementary Information Supplementary Information for Dimensional crossover and cold-atom realization of topological Mott insulators
