# Supplementary material for: Dimensional crossover and cold-atom realization of topological Mott insulators
Source: Sci Rep. 2015 Feb 11;5:8386. doi: 10.1038/srep08386 (PMC4323638; doi:10.1038/srep08386)
Supplement: Supplementary Information — for Dimensional crossover and cold-atom realization of topological Mott insulators [file srep08386-s1.pdf]

Supplementary Information for "Dimensional crossover and cold-atom realization of topological Mott insulators"

Mathias S. Scheurer,[1] Stephan Rachel,[2] and Peter P. Orth[1]

[1]*Institute for Theory of Condensed Matter, Karlsruhe Institute of Technology (KIT), 76131 Karlsruhe, Germany*

[2]*Institute for Theoretical Physics, TU Dresden, 01062 Dresden, Germany*

(Dated: December 12, 2014)

SUPPLEMENTARY FIGURES

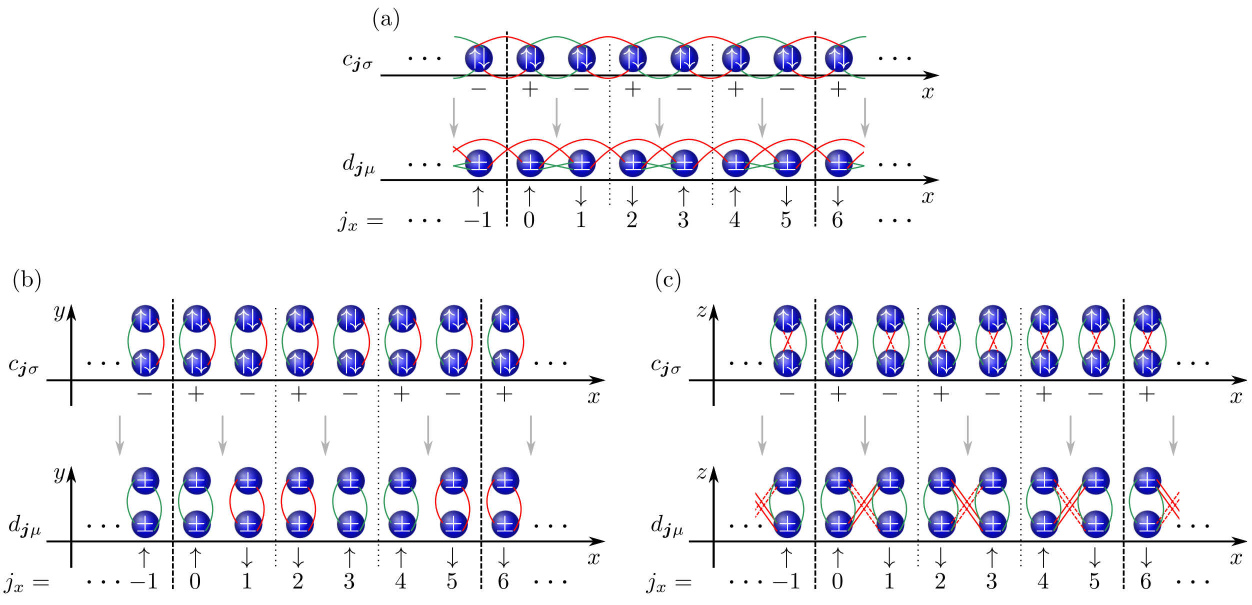

Supplementary Figure S1. **Graphical definition of the transformation to the new lattice and illustration of the required hopping matrix elements along the three different directions.** Panels (a, b, c) refer to hopping elements along the (x, y, z) spatial directions. The upper part of the respective panels refers to the hopping elements of the $c_{\boldsymbol{j}\sigma}$ fermions in the Hamiltonian $H_{2D} + H_z$ defined in Eqs. (1) and (2) of the main text. The lower part of the panels shows the required hopping elements of the $d_{\boldsymbol{j}\mu}$ fermions that have to be implemented in order to obtain the (identical) Hamiltonian $H_{2D} + H_z$ in terms of the $c_{\boldsymbol{j}\sigma}$ fermions if the spin σ is encoded spatially (as even and odd sites) and the site parity along x, $\mu = \pm$, as the internal hyperfine degree of the freedom.

SUPPLEMENTARY DISCUSSION

S1. Ambiguity of the term "Topological Mott Insulator"

In the literature, the term "Topological Mott insulator" is commonly used for two phenomena or states of matter which have nothing in common except that they belong to the very broad field of topological phases.

(i) One was introduced by Sri Raghu and collaborators [1]: they considered electrons on the honeycomb lattice in the presence of (strong) nearest and next-nearest neighbor repulsive interactions. They argued that the second-neighbor repulsions might generate an effective spin-orbit term via spontaneous symmetry breaking leading to quantum anomalous Hall and quantum spin Hall phases. The *topological Mott insulator* of Raghu *et al.* is topologically equivalent to the two-dimensional topological insulator, and the additional word "Mott" only emphasizes that the non-trivial topology is induced by virtue of interactions. Several authors have studied this type of system recently [2–7]. In principle, also the topological Kondo insulator falls into this category [8, 9]. It is important to emphasize that this type of topological Mott insulator is effectively described by a non-interacting topological insulator model.

(ii) The other one was introduced by Dima Pesin and Leon Balents [10] and has been considered in this paper. As motivated in the paper, the *topological Mott insulator* phase of Pesin and Balents is a spin liquid state where the charge and spin degrees of freedom have been separated; the charge undergoes a Mott insulator transition while the deconfined spinons inherit the non-trivial band topology of the topological insulator band structure. With other words, the charge degree forms a *Mott* insulator, while the spin degree is a *topological* insulator, both together are, hence, named *topological Mott insulator*. Recently, this state of matter was also investigated by other authors [11–13]. It is important to emphasize that this type of topological Mott insulator merely exists because of the interplay of non-trivial band topology and strong interactions. It is a fractionalized phase which does *not* have any non-interacting analogue.

S2. Twisting the Hubbard interaction via permutation of the degrees of freedom

In the following we present more details about how the non-local interaction term

$$H'_U = \frac{U}{2} \sum_{\boldsymbol{j},\sigma=\uparrow,\downarrow} (n_{2j_x j_y j_z \sigma} + n_{(2j_x+1) j_y j_z \sigma} - 1)^2, \quad n_{\boldsymbol{j}\sigma} = c^\dagger_{\boldsymbol{j}\sigma} c_{\boldsymbol{j}\sigma}, \tag{S1}$$

considered in this work can be implemented in a cold-atom setup. The procedure we propose is based on interchanging degrees of freedom: As illustrated in Supplementary Fig. S1, the spin-degree of freedom of the original fermions $c_{\boldsymbol{j}\sigma}$ will be encoded by the evenness and oddness of j_x of the new fermions $d_{\boldsymbol{j}\mu}$, whereas the parity $(-1)^{j_x}$ of j_x in the original basis is represented by the internal onsite degree of freedom $\mu = \pm$ of the new fermions. By construction, the nonlocal interaction term in Eq. (S1) assumes the form of the usual Hubbard onsite interaction in terms of the transformed fermions $d_{\boldsymbol{j}\mu}$. Keeping the non-interacting part of the theory $H_{2\mathrm{D}} + H_z$ fixed, the full Hamiltonian reads in the new basis

$$\begin{aligned} H = &- \sum_{\boldsymbol{j},\boldsymbol{j}'} \sum_{\mu,\mu'} \left(d^\dagger_{\boldsymbol{j}\mu} \mathcal{A}_{\mu,\mu'}(\boldsymbol{j},\boldsymbol{j}') d_{\boldsymbol{j}'\mu'} + \mathrm{H.c.} \right) + \lambda \sum_{\boldsymbol{j}} \sum_{\mu,\mu'} d^\dagger_{\boldsymbol{j}\mu} \left(\sigma_z\right)_{\mu,\mu'} d_{\boldsymbol{j}\mu'} \\ &+ \frac{U}{2} \sum_{\boldsymbol{j}} \left(\sum_{\mu} d^\dagger_{\boldsymbol{j}\mu} d_{\boldsymbol{j}\mu} - 1 \right)^2, \end{aligned} \tag{S2}$$

where $\mathcal{A}_{\mu,\mu'}(\boldsymbol{j},\boldsymbol{j}')$ are the transformed hopping amplitudes of $H_{2\mathrm{D}} + H_z$ as illustrated in Supplementary Fig. S1 for all three spatial directions. Redistributing the original spin degree of freedom in the way we propose, the system only contains nearest and next-nearest neighbor hopping elements. It is very important to note that, although the staggered hopping now has the form of a Zeeman term, Eq. (S2) is mathematically equivalent to $H_{2\mathrm{D}} + H_z + H'_U$ and, consequently, respects time-reversal symmetry.

---

[1] Raghu, S., Qi, X.-L., Honerkamp, C. & Zhang, S.-C. Topological Mott insulators. *Phys. Rev. Lett.* **100**, 156401 (2008).

[2] Weeks, C. & Franz, M. Interaction-driven instabilities of a dirac semimetal. *Phys. Rev. B* **81**, 085105 (2010).

[3] Uebelacker, S. & Honerkamp, C. Instabilities of quadratic band crossing points. *Phys. Rev. B* **84**, 205122 (2011).

[4] Dauphin, A., Müller, M. & Martin-Delgado, M. A. Rydberg-atom quantum simulation and chern-number characterization of a topological mott insulator. *Phys. Rev. A* **86**, 053618 (2012).

[5] Daghofer, M. & Hohenadler, M. Phases of correlated spinless fermions on the honeycomb lattice. *Phys. Rev. B* **89**, 035103 (2014).

[6] Garcia-Marinez, N. A., Grushin, A. G., Neupert, T., Valenzuela, B. & Castro, E. V. Interaction-driven phases in the half-filled spinless honeycomb lattice from exact diagonalization. *Phys. Rev. B* **88**, 245123 (2013).

[7] Duric, T., Chancellor, N. & Herbut, I. F. Interaction-induced anomalous quantum hall state on the honeycomb lattice. *Phys. Rev. B* **89**, 165123 (2014).

[8] Dzero, M., Sun, K., Galitski, V. & Coleman, P. Topological kondo insulators. *Phys. Rev. Lett.* **104**, 106408 (2010).

[9] Neupane, M. & *et al.* Surface electronic structure of the topological Kondo insulator candidate correlated electron system SmB6. *Nature Comm.* **04**, 2991 (2013).

[10] Pesin, D. A. & Balents, L. Mott physics and band topology in materials with strong spin-orbit interaction. *Nature Phys.* **6**, 376 (2010).

[11] Witczak-Krempa, W., Choy, T. P. & Kim, Y. B. Gauge field fluctuations in three-dimensional topological mott insulators. *Phys. Rev. B* **82**, 165122 (2010).

[12] Rachel, S. & Le Hur, K. Topological insulators and mott physics from the hubbard interaction. *Phys. Rev. B* **82**, 075106 (2010).

[13] Kargarian, M., Wen, J. & Fiete, G. A. Competing exotic topological insulator phases in transition-metal oxides on the pyrochlore lattice with distortion. *Phys. Rev. B* **83**, 165112 (2011).
